# Panton-Valentine Leukocidin Is Not the Primary Determinant of Outcome for *Staphylococcus aureus* Skin Infections: Evaluation from the CANVAS Studies

**DOI:** 10.1371/journal.pone.0037212

**Published:** 2012-05-18

**Authors:** Amy Tong, Steven Y. C. Tong, Yurong Zhang, Supaporn Lamlertthon, Batu K. Sharma-Kuinkel, Thomas Rude, Sun Hee Ahn, Felicia Ruffin, Lily Llorens, Ganesh Tamarana, Donald Biek, Ian Critchley, Vance G. Fowler

**Affiliations:** 1 Division of Infectious Diseases, Department of Medicine, Duke University Medical Center, Durham, North Carolina, United States of America; 2 Duke Clinical Research Institute, Durham, North Carolina, United States of America; 3 Naresuan University, Phitsanulok, Phitsanulok Province, Thailand; 4 Menzies School of Health Research, Darwin, Northern Territory, Australia; 5 Cerexa, Inc., Oakland, California, United States of America; Rockefeller University, United States of America

## Abstract

The impact of Panton-Valentine leukocidin (PVL) on the severity of complicated skin and skin structure infections (cSSSI) caused by *Staphylococcus aureus* is controversial. We evaluated potential associations between clinical outcome and PVL presence in both methicillin-resistant *S. aureus* (MRSA) and methicillin-susceptible *S. aureus* (MSSA) isolates from patients enrolled in two large, multinational phase three clinical trials assessing ceftaroline fosamil for the treatment of cSSSI (the CANVAS 1 and 2 programs). Isolates from all microbiologically evaluable patients with monomicrobial MRSA or MSSA infections (n = 473) were genotyped by PCR for *pvl* and underwent pulsed-field gel electrophoresis (PFGE). Genes encoding *pvl* were present in 266/473 (56.2%) isolates. Infections caused by *pvl*-positive *S. aureus* were associated with younger patient age, North American acquisition, and presence of major abscesses (*P*<0.001 for each). Cure rates of patients infected with *pvl-*positive and *pvl-*negative *S. aureus* were similar overall (93.6% versus 92.8%; *P* = 0.72), and within MRSA-infected (94.5% vs. 93.1%; *P* = 0.67) and MSSA-infected patients (92.2% vs. 92.7%; *P* = 1.00). This finding persisted after adjustment for multiple patient characteristics. Outcomes were also similar when USA300 PVL+ and non-USA300 PVL+ infections were compared. The results of this contemporary, international study suggest that *pvl* presence was not the primary determinant of outcome in patients with cSSSI due to either MRSA or MSSA.

## Introduction

*Staphylococcus aureus* is the leading cause of complicated skin and skin structure infections (cSSSI) in the United States [1]–[4]. A number of studies have reported a high prevalence of Panton-Valentine leukocidin (PVL), a pore-forming, bi-component toxin that causes neutrophil lysis [5], among bacterial isolates causing cSSSI [6]. While the association between cSSSI and presence of *pvl* is particularly strong with community-acquired methicillin-resistant *S. aureus* (MRSA) [7], it has also been described among methicillin-susceptible *S. aureus* (MSSA) isolates causing cSSSI [3], [8].

The role of PVL in determining the outcome and severity of *S. aureus* cSSSI remains one of the most controversial topics in the field of staphylococcal research [9]. The presence of *pvl* in *S. aureus* strains has been associated with severe infections in some [10]–[15] but not all [9], [16], [17] previous studies. For example, our group has reported that *pvl* presence was paradoxically associated with a better clinical outcome than cSSSI infections caused by *pvl*-negative MRSA [17], [18]. For this reason, the role of *pvl* in cSSSI is unresolved.

The goal of the current study was to evaluate the impact of *pvl* presence on the outcome of patients with cSSSI caused by both MRSA and MSSA. To accomplish this goal, we used clinically well-characterized *S. aureus* isolates collected from patients with cSSSI who were enrolled in two phase 3 multinational clinical trials. The objectives of the current study were: 1) to confirm our previous observations that *pvl* is not the primary determinant of clinical outcome in patients with cSSSI due to MRSA, and 2) to test whether this same finding is also encountered in MSSA-infected patients. In addition, we aimed to evaluate whether outcomes of infections caused by USA300 PVL+ isolates differed from those caused by non-USA300 PVL+ isolates.

## Methods

### Patients and settings

CANVAS 1 (NCT00424190) and CANVAS 2 (NCT00423657; Ceftaroline versus Vancomycin in Skin and Skin-Structure Infection) were two methodologically identical, randomized, double-blind, multinational phase 3 clinical trials that compared the efficacy and safety of intravenous ceftaroline fosamil monotherapy with intravenous vancomycin plus aztreonam combination therapy for the treatment of adults with cSSSI caused by gram-positive and gram-negative organisms. The study designs of the CANVAS trials have been published in detail [19]–[21].

A total of 1378 patients from 111 participating centers in 12 countries were enrolled from February 2007 through December 2007 [19], [20]. Men and non-pregnant women aged ≥18 years and diagnosed with cSSSI severe enough to require initial hospitalization or treatment in an emergency department and ≥5 days of intravenous antimicrobial therapy and that involved either (1) deep soft tissue or required deep surgical intervention, or (2) cellulitis or abscess of a lower extremity in patients with diabetes mellitus (DM) or well-documented peripheral vascular disease (PVD), were eligible for the study. Purulent or seropurulent drainage and/or collection or the presence of at least three of the following signs or symptoms was also required for participation: erythema, fluctuance, heat and/or localized warmth, pain and/or tenderness to palpation, fever (temperature >38°C) or hypothermia, white blood cell (WBC) count of >10,000 / mm^3^, or >10% immature neutrophils irrespective of WBC count.

Patients were randomized to receive either ceftaroline fosamil (600 mg every 12 h) followed by normal saline placebo or vancomycin followed by aztreonam (1 g each every 12 h) for 5–14 days. Bacterial isolates were obtained from all patients at baseline by needle aspiration or a surgical procedure. Test-of-cure evaluation was performed 8 to 15 days after administration of the last dose of the study drug. The clinical response at the test of cure was classified as cure, failure, or indeterminate [21]. Clinical cure was defined as total resolution of all signs and symptoms of the baseline infection or improvement such that no further antimicrobial therapy was necessary. The microbiologically evaluable (ME) population was defined as clinically evaluable patients with ≥1 bacterial pathogen isolated from blood or the cSSSI site at baseline, excluding patients whose baseline cultures revealed monomicrobial *Pseudomonas aeruginosa* or anaerobic infection. In the present investigation, the analysis population was defined as microbiologically evaluable patients with cSSSI due to monomicrobial *S. aureus* and a clinical response at test of cure evaluation. The investigation was approved by the Duke University Medical Center Institutional Review Board.

### Pulsed Field Gel Electrophoresis

Pulsed-field gel electrophoresis (PFGE) with the restriction enzyme *SmaI* was performed as previously described [22]. The PFGE profiles were analyzed using BioNumerics software (Applied Maths, Kortrijk, Belgium). A similarity coefficient of 80% was used to define pulsed-field type clusters. Dice coefficients (pairwise similarity) were calculated for each pair of isolates, and a dendrogram was constructed using an optimization value of 0.50% and a position tolerance ranging from 1.25% to 1.35% (the end of the fingerprint). PFGE profiles were interpreted according to a published typing schema [22].

### PCR assays for genotyping

*S. aureus* genomic DNA was extracted using an ultraclean microbial DNA kit (MO BIO Laboratories, Inc., Carlsbad, CA) in accordance with the manufacturer's instructions. PCR assays were used to screen the *S. aureus* genome for the putative bacterial virulence toxin *pvl* for all *S. aureus* isolates using previously described methods [17], [18].

### Statistical methods

Cure rate by *pvl* status was stratified by markers of severity, including abscess versus non-abscess infection type, presence of fever, white blood cells (>10,000/ mm^3^), infection size (baseline infection area of >100 cm^2^), presence of diabetes, patient age, and study medication. Infection size was calculated as the product of the length and width of the primary infection site at its longest and widest axes, respectively. Continuous variables were compared between groups by using the two-sample *t* test. Categorical variables were analyzed using Fisher's exact test, its 2-by-K extension, or the Cochran-Mantel-Haenszel test, as appropriate, for stratified analyses. Our *a priori* hypothesis, that the presence of *pvl* in MRSA cSSSI is not associated with a worse clinical outcome, was tested using Fisher's exact test. All reported *P* values are two sided. *P* values of <0.05 were deemed statistically significant. Since subjects were not randomized on the basis of *pvl* status, all *P* values calculated should be considered descriptive and not inferential. Results were obtained using SAS software, version 9.1.3 (SAS Institute Inc, Cary, NC, USA).

## Results

### Study population and baseline characteristics

A total of 473 patients from the CANVAS studies with cSSSI due to monomicrobial *S. aureus* (280 MSSA, 59.2%; 193 MRSA, 40.8%) and for whom clinical outcome were available, were included in the current study. Among these 473 study patients, 253 (53.5%) received ceftaroline fosamil and 220 (46.5%) received vancomycin plus aztreonam. Patients were predominantly white, male, and North American, with an average age of 45 years. The majority of infections were either major abscesses (202 [42.7%]) or deep/extensive cellulitis (144 [30.4%]) (Table 1).

**Table 1:** Baseline characteristics of 473 patients with *S. aureus* complicated skin and skin structure infections (cSSSI) stratified by methicillin-resistance and *pvl* gene status of the infecting pathogen.

	*Value for indicated patient group; n(%) except where noted*					
	*MRSA (n = 193)*			*MSSA (n = 280)*		
*Parameter*	*pvl-*negative *(n = 29)*	*pvl-*positive* (n = 164)*	*P-Value* *	*pvl-*negative* (n = 178)*	*pvl-*positive* (n = 102)*	*P-Value* *
Demographic characteristics						
Mean age(yr) ± SD	53.1±17.2	40.6±14.0	<0.0001**	51.2±15.1	40.2±16.0	<0.0001**
Male	15 (51.7%)	96 (58.5%)	0.5440	119 (66.9%)	55 (53.9%)	0.0403
White race	27 (93.1%)	94 (57.3%)	0.0053	148 (83.1%)	87 (85.3%)	0.2357
Source of infection			<0.0001̂			<0.0001̂
Infected Wound	1 (3.4%)	19 (11.6%)		30 (16.9%)	6 (5.9%)	
Major Abscess	4 (13.8%)	95 (57.9%)		46 (25.8%)	57 (55.9%)	
Infected Ulcer	11 (37.9%)	0 (0.0%)		11 (6.2%)	2 (2.0%)	
Infected Burn	10 (34.5%)	2 (1.2%)		6 (3.4%)	0 (0.0%)	
Infected Bite	0 (0.0%)	9 (5.5%)		1 (0.6%)	3 (2.9%)	
Deep/Extensive Cellulitis	2 (6.9%)	36 (22.0%)		72 (40.4%)	34 (33.3%)	
Lower Extremity SSSI in Diabetic Subject	1 (3.4%)	3 (1.8%)		7 (3.9%)	0	
Geographical location			<0.0001^§^			0.0046^§^
Eastern Europe	20 (69.0%)	4 (2.4%)		94 (52.8%)	67 (65.7%)	
Latin America	2 (6.9%)	3 (1.8%)		18 (10.1%)	1 (1.0%)	
Western Europe	2 (6.9%)	0 (0.0%)		24 (13.5%)	8 (7.8%)	
US	5 (17.2%)	157 (95.7%)		42 (23.6%)	26 (25.5%)	
Prior antibiotic use			<0.0001±			0.2323±
No prior therapy	22 (75.9%)	52 (31.7%)		121 (68.0%)	59 (57.8%)	
≤24 hours of treatment	6 (20.7%)	84 (51.2%)		45 (25.3%)	34 (33.3%)	
>24 hours of treatment	1 (3.4%)	28 (17.1%)		12 (6.7%)	9 (8.8%)	
MRSA risk factors						
Hospitalization	27 (93.1%)	73 (44.5%)	<0.0001	156 (87.6%)	85 (83.3%)	0.3704
Antibiotic use in 4 weeks prior	16 (55.2%)	111 (67.7%)	0.2071	62 (34.8%)	38 (37.3%)	0.6990
Diabetes Mellitus	4 (13.8%)	20 (12.2%)	0.7642	43 (24.2%)	7 (6.9%)	0.0003
Peripheral Vascular Disease	9 (31.0%)	2 (1.2%)	<0.0001	24 (13.5%)	5 (4.9%)	0.0250
Infection characteristics						
Fever (temp of >38°C)^1^	19 (65.5%)	14 (8.6%)	<0.0001	69 (38.8%)	43 (42.2%)	0.6131
White Blood Cells >10,000 /mm^3^ **, 1	9 (34.6%)	72 (46.2%)	0.2954	49 (30.4%)	34 (41.5%)	0.1152
Baseline infection area of >100 cm^2^ **	22 (75.9%)	101 (61.6%)	0.2079	121 (68.0%)	60 (58.8%)	0.1529
Duration of antibiotic treatment (days median) ***	8.0	9.5	0.1836	7.0	7.0	0.0054
Study medication			0.5401			1.0000
Ceftaroline	19 (65.5%)	94 (57.3%)		89 (50.0%)	51 (50.0%)	
Vancomycin plus Aztreonam	10 (34.5%)	70 (42.7%)		89 (50.0%)	51 (50.0%)	

* = P-Value from generalized 2-sided Fishers Exact Test. Calculated using 7×2 table (^∧^), 4×2 table (§) and 3×2 table (±).

** = P-Value from 2-sided T-test.

*** = P-value from Wilcoxon Rank Sum test.

1 = Subjects with missing data were not included in these summaries.

### Patient characteristics according to *pvl* presence and methicillin-susceptibility

Overall, *pvl* was detected in 266/473 (56.2%) of the *S. aureus* isolates (Table 1; MRSA 164/193 [85.0%], MSSA 102/280 [36.4%]). In both the MRSA-infected and MSSA-infected cohorts, presence of *pvl* was associated with younger age, and major abscess (*P*<0.001). Patients with *pvl-*positive MRSA were significantly more likely to be from North America, and less likely to have been hospitalized or have PVD (all *P*<0.001) than patients with *pvl*-negative MRSA infections. Patients with *pvl-*positive MSSA were less likely to have other comorbid conditions, including DM (*P*<0.001) or PVD (*P* = 0.03) than patients with *pvl*-negative MSSA infections.

### Association of *pvl* gene status and patient clinical outcome

Overall, cure rates among patients with cSSSI due to *pvl*-positive and *pvl*-negative *S. aureus* did not differ significantly (*pvl*-positive *S. aureus* 93.6% [249/266] vs. *pvl*-negative *S. aureus* 92.8% [192/207]; *P* = 0.72). Cure rates remained similar when stratified by methicillin-resistance (Table 2). Similar findings also persisted in both the MRSA and MSSA populations after stratification by infection type (abscess versus non-abscess), presence of fever, leukocytosis, infection size (baseline infection area of >100 cm^2^), patient age, or DM (Table 2).

**Table 2:** Clinical outcome of cure for 473 patients with methicillin-resistant and methicillin-susceptible *S. aureus* complicated skin and skin structure infections by *pvl* gene status, stratified by infection severity, diabetes, patient age, and study medication.

	No. of cured patients/total no. of patients (%) with indicated status					
	MRSA (n = 193)			MSSA (n = 280)		
Covariate	pvl-negative (n = 29)	pvl-positive (n = 164)	P-Value*	pvl-negative (n = 178)	pvl-positive (n = 102)	P-Value*
Overall	27/29 (93.1%)	155/164 (94.5%)	0.6722	165/178 (92.7%)	94/102 (92.2%)	1.0000
Type of infection						
Abscess	3/4 (75.0%)	90/95 (94.7%)	0.2244	42/46 (91.3%)	53/57 (93.0%)	1.0000
Non-abscess	24/25 (96.0%)	65/69 (94.2%)	1.0000	123/132 (93.2%)	41/45 (91.1%)	0.7412
Baseline fever (temp >38°C)	19/19 (100.0%)	13/14 (92.9%)	0.4242	65/69 (94.2%)	40/43 (93.0%)	1.0000
White blood cells >10,000 / mm^3^	7/9 (77.8%)	68/72 (94.4%)	0.1306	45/49 (91.8%)	30/34 (88.2%)	0.7107
Baseline infection area of >100 cm^2^	21/22 (95.5%)	95/101 (94.1%)	1.0000	113/121 (93.4%)	59/60 (98.3%)	0.2753
Diabetes	3/4 (75.0%)	18/20 (90.0%)	0.4368	39/43 (90.7%)	7/7 (100.0%)	1.0000
Patient age (<65 y)	18/19(94.7%)	150/159(94.3%)	1.0000	136/145 (93.8%)	84/92 (91.3%)	0.6065
Study medication						
Ceftaroline	17/19 (89.5%)	91/94 (96.8%)	0.1962	82/89 (92.1%)	46/51 (90.2%)	0.7578
Vancomycin plus Aztreonam	10/10 (100.0%)	64/70 (91.4%)	1.0000	83/89 (93.3%)	48/51 (94.1%)	1.0000

* = P-Value from generalized 2-sided Fishers Exact Test.

Missing categories are not in the analyses.

### PFGE profiles

A total of 470 isolates successfully underwent PFGE genotyping (three isolates failed *smaI* digestion). Of these 470 isolates, 231 (49.1%) were typeable by the USA typing schema. Among the typeable isolates, 171 (74.0%) were USA300. Other strain types identified included USA100 (2 isolates; 0.9%), USA200 (19 isolates; 8.2%); USA400 (6 isolates; 2.6%); USA600 (29 isolates; 12.6%); USA700 (1 isolate; 0.4%); and USA800 (3 isolates; 1.3%).

A comparison of infections caused by USA300 PVL+ with non-USA300 PVL+ isolates (Table 3) demonstrated that USA300 PVL+ infections were associated with non-Caucasian ethnicity, North America, prior antibiotic use, MRSA, and the absence of hospitalization as a risk factor (all *P*<0.001). Neither the presence of a deep abscess, the receipt of surgical intervention nor the antibiotic regimen received differed between the two groups. Outcomes from USA300 PVL+ and non-USA300 PVL+ infections were similar overall (*P* = 0.80) and when stratified across multiple sub-groups including methicillin-susceptibility (Table 4).

**Table 3:** Baseline characteristics of 266 patients with *pvl* positive *S. aureus* complicated skin and skin structure infections (cSSSI) stratified by pulsed-field gel electrophoresis type.

	*Value for indicated patient group; n(%) except where noted*		
*Parameter*	*USA300 (n = 162)*	*Non-USA300 (n = 104)*	*P-Value* *
Demographic characteristics			
Mean age(yr)± SD	40.1 (13.9)	41.1 (16.1)	0.6012
Male	90 (55.6)	61 (58.7)	0.7037
White race	94 (58.0)	87 (83.7)	<0.0001
Source of infection			0.0283^^^
Infected Wound	16 (9.9)	9 (8.7)	
Major Abscess	99 (61.1)	53 (51.0)	
Infected Ulcer	0 (0)	2 (1.9)	
Infected Burn	0 (0)	2 (1.9)	
Infected Bite	9 (5.6)	3 (2.9)	
Deep/Extensive Cellulitis	35 (21.6)	35 (33.7)	
Lower Extremity SSSI in Diabetic Subject	3 (1.9)	0 (0)	
Geographical location			<0.0001^§^
Eastern Europe	1 (0.6)	70 (67.3)	
Latin America	0 (0)	4 (3.8)	
Western Europe	0 (0)	8 (104)	
US	161 (99.4)	22 (21.2)	
Prior antibiotic use			<0.0001±
No prior therapy	49 (30.2)	62 (59.6)	
≤24 hours of treatment	84 (51.9)	34 (32.7)	
>24 hours of treatment	29 (17.9)	8 (7.7)	
MRSA risk factors			
Hospitalization	69 (42.6)	89 (85.6)	<0.0001
Antibiotic use in 4 weeks prior	108 (66.7)	41 (39.4)	<0.0001
Diabetes Mellitus	18 (11.1)	9 (8.7)	0.6779
Peripheral Vascular Disease	2 (1.2)	5 (4.8)	0.1144
Infection characteristics			
Fever (temp of >38°C)	13 (8.0)	44 (42.3)	<0.0001
White Blood Cells >10,000 / mm^3^ **,	76 (46.9)	30 (28.8)	0.1006
MRSA	141 (87)	23 (22.1)	<0.0001
Surgical intervention	115 (71.0)	64 (61.5)	0.7919
Study medication			0.5294
Ceftaroline	91 (56.2)	54 (51.9)	
Vancomycin plus Aztreonam	71 (43.8)	50 (48.1)	

* = P-Value from generalized 2-sided Fishers Exact Test. Calculated using 7×2 table (^), 4×2 table (§) and 3×2 table (±).

** = P-Value from 2-sided T-test.

**Table 4:** Clinical outcome of cure for 266 patients with *pvl* positive *S. aureus* complicated skin and skin structure infections by pulsed-field gel type, stratified by infection severity, diabetes, patient age, methicillin-susceptibility and study medication.

	No. of cured patients/total no. of patients (%) with indicated status		
Covariate	USA300 *(n = 162*)	Non-USA300 *(n = 104)*	*P-Value* *
Overall	151/162 (93.2)	98/104 (94.2)	0.8030
Type of infection			
Abscess	96/101 (95.0)	49/53 (92.5)	0.4954
Infection wound	13/16 (81.3)	8/9 (88.9)	1.0000
Other non-abscess	42/45 (93.3)	41/42 (97.6)	0.6171
Baseline fever (temp >38°C)	12/13 (92.3)	41/44(93.2)	1.0000
White blood cells >10,000 /mm^3^	70/76 (92.1)	28/30 (93.3)	1.0000
Baseline infection area of>100 cm^2^	92/98 (93.9)	62/63 (98.4)	0.2478
Diabetes	16/18 (88.9)	9/9 (100.0)	0.5385
Patient age (<65 y)	147/158 (93.0)	87/93 (93.5)	1.0000
Surgical intervention**	109/115 (94.8)	60/64 (93.8)	0.7466
MRSA	132/141 (93.6)	23/23 (100.0)	0.3623
Study medication			
Ceftaroline	87/91 (95.6)	50/54 (92.6)	0.4707
Vancomycin plus Aztreonam	64/71 (90.1)	48/50 (96.0)	0.3039

* = P-Value from generalized 2-sided Fishers Exact Test.

** Receipt of a surgical procedure ≤48 hours post enrolment.

Missing categories are not in the analyses.

## Discussion

The significance of PVL in the outcome of staphylococcal infections remains unresolved. The present study used a large, contemporary, multinational collection of MRSA and MSSA isolates to evaluate the impact of *pvl* presence on clinical outcome of patients with cSSSI. Despite adjustment for a number of clinically relevant variables, *pvl* presence was not associated with a higher risk of failure. This finding has several implications.

This analysis of the isolates collected in the CANVAS 1 and 2 studies add to an increasing body of evidence [17], [18], [23] that the simple presence of *pvl* is not the primary outcome determinant in all *S. aureus* infections. This finding persisted after adjusting for patient comorbidities, infection type, infection severity, and patient age. The results of this study are consistent with two previous reports by our group evaluating potential associations between *pvl* and cSSSI caused by MRSA [17], [18]. However, the current investigation extends our understanding of the role of *pvl* in staphylococcal pathogenesis by considering its relevance in MSSA infections. While *pvl* is currently considered primarily in the setting of MRSA infections [1]–[4], its presence in MSSA is also well established [3], [6], [8], [24]–[26]. Collectively, these results underscore the recent conclusions of Otto [9] that factors other than the mere presence of *pvl* are the primary determinants of clinical outcome in patients with *S. aureus* cSSSI.

However, the infections process is a continuum from onset to resolution and while outcomes did not differ, the presence of *pvl* was associated with important clinical characteristics in patients with *S. aureus* cSSSI. Patients with *pvl*-positive cSSSI were younger, more likely to have major abscess, more likely to be North American and less likely to have health care contact, findings that have been previously noted [17], [26]. In contrast, patients with *pvl*-negative strains tended to be older and have comorbidities such as diabetes or peripheral vascular disease. These differences persisted in both the MRSA and MSSA subgroups, and may reflect in part the epidemiologic settings in which the infections were acquired. Healthcare-associated *S. aureus* infections are less likely to contain *pvl* [2] and are also more likely to cause cSSSI in older patients with more comorbid conditions. By contrast, presence of *pvl* is often associated with community-acquired infection. Thus, the presence of *pvl* is associated with a different spectrum of disease in a distinct subgroup of patients and also influences the treatment received in that *pvl*+ cSSSI more frequently required surgical management. However, the need for surgical intervention is a clinical decision and prior knowledge of *pvl* status is unlikely to be of assistance at the time this decision is made.

Following the provision of appropriate management, the current investigation underscores the fact that *pvl* is not the principle determinant of clinical outcome in patients with either MRSA or MSSA cSSSI. Factors other than *pvl* should be considered for future therapeutic and diagnostic strategies in combating *S. aureus* cSSSI. For example, other characteristics including alpha hemolysin [27], a new bicomponent leukotoxin [28], the antagonistic effects of antibody to control proliferation of PVL-producing *S. aureus* [29], and host species specificity of the neutrophil lysis properties of PVL [30], may also be associated with the clinical outcome of *S. aureus* cSSSI. It is also possible that the genes coding for PVL may simply be markers of particular bacterial clones and are not themselves directly involved in the pathogenesis of more severe infection.

Due to the predominance of USA300 in North America as previously described and also in this study, most reports regarding PVL from this region focus on this strain of *S. aureus*. The multinational nature of the CANVAS studies allowed us to compare infections caused by USA300 PVL+ with non-USA300 PVL+ isolates. Despite some differences in epidemiology regarding the likelihood of community-acquisition, deep abscesses were the main clinical manifestation of both groups and, importantly, the need for surgery and outcomes were equivalent. This suggests that findings relating to the clinical manifestations and outcomes of cSSSI due to USA300 are likely to be generalizable to other PVL+ infections. However, the epidemic spread of USA300 in North America compared to PVL+ clones elsewhere in the world remains unexplained and our data indicate that factors other than PVL may play a more important role in the success of USA300.

The *S. aureus* isolates collections used in this study are large, contemporary, multinational, cSSSI specific, and clinically characterized to the level of rigor of a U.S. Food and Drug Administration (FDA) registrational trial. Limitations include the fact that we did not evaluate quantitative *pvl* expression or the presence of *pvl* polymorphisms, as these properties may influence the function of gene products [31], [32], although *pvl* polymorphisms have not been found to be of clinical importance [26]. By design, this investigation focused only upon infections in which the causative pathogen was available for culture. We were also unable to consider potential relationships between vancomycin trough levels and outcome.

In summary, this study has demonstrated that presence of *pvl* in *S. aureus* strains is not associated with a worse outcome in patients with cSSSI. This finding persisted regardless of methicillin-resistance status. These findings indicate that factors other than the presence of *pvl* are the primary determinants of clinical outcome in *S. aureus* cSSSI. Future efforts are necessary for a more complete understanding of the role of *pvl* in the pathogenesis of serious infections due to *S. aureus*.
